# Type 2 diabetes mellitus, physical activity, exercise self-efficacy, and body satisfaction. An application of the transtheoretical model in older adults

**DOI:** 10.1080/21642850.2014.924858

**Published:** 2014-07-15

**Authors:** Marco Guicciardi, Romina Lecis, Chiara Anziani, Lucina Corgiolu, Adele Porru, Matteo Pusceddu, Francesca Spanu

**Affiliations:** ^a^ Department of Pedagogy, Psychology, Philosophy, University of Cagliari, Via Is Mirrionis, 1, 09123Cagliari, Italy; ^b^ Group Physical Activity Project, Centre of Diabetology, San Giovanni University Hospital, Via Ospedale 46, 09124Cagliari, Italy

**Keywords:** physical activity, diabetes, elderly, self-efficacy, body satisfaction

## Abstract

Physical activity (PA) is a relevant component of the treatment of Type 2 diabetes mellitus (T2DM). However, to prevent its related morbidities, PA requires an immediate and lasting change of lifestyle. Exercise self-efficacy and body satisfaction were used in a sample of older adults with T2DM, classified in different stages of change, to predict levels of PA. Results show that exercise self-efficacy increases linearly from precontemplation to maintenance stage, while body satisfaction shows an inverted U shape. However, only stages of change, other than exercise self-efficacy, add a significant and noticeable contribution to prediction of levels of PA. This evidence claims a tailored approach to PA in older adults with T2DM and advises behavioural health interventions based on exercise self-efficacy.

This research takes into account the transtheoretical model (TTM), exercise self-efficacy, and body satisfaction to investigate some determinants of physical activity (PA) in people with Type 2 diabetes mellitus (T2DM).

PA can benefit people with Type 2 diabetes and prevent its related morbidities. PA improves insulin sensitivity and blood glucose control, reduces the risk of cardiovascular disease (CVD), increases balance and coordination, enhances energy levels and endurance, and has beneficial effects on the control of factors related to diabetes (hypertension, lipid profile, and weight loss) (Tuomiletho et al., 2001).

The large Italian Diabetes and Exercise Study has demonstrated that twice-weekly supervised, facility-based, aerobic, and resistance training on top of exercise counselling is superior to counselling alone in promoting PA, improving physical fitness, haemoglobin (Hb) A_1c_, and CVD risk profile, and reducing medication number and/or dosage in sedentary patients with Type 2 diabetes (Balducci et al., 2012).

Prior research has demonstrated a positive relationship between self-efficacy and self-care behaviours (Williams & Bond, 2002), active lifestyles (Hall & McAuley, 2010), and exercise behaviour in T2DM populations (Aljasem, Peyrot, Wissow, & Rubin 2001; Sarkar, Fisher, & Schillinger, 2006). Self-efficacy beliefs influence the choice of activities, the level of effort, the perseverance in spite of difficulties, the cognitive evaluations, and emotional reactions after successes and failures (Bandura, 1997).

Exercise self-efficacy is an important predictor of the adoption and maintenance of PA. The contribution of exercise self-efficacy has been examined in a variety of clinical and disability populations, including those with cancer, stroke, spinal cord injury, and diabetes (Kirk, MacMillan, & Webster, 2010; Kroll et al., 2012).

Also body satisfaction has been found significantly associated with the motives underlying adherence to PA, especially in overweight or obese persons (Ferrand, Perrin, & Nasarre, 2008; Grogan, 2006; Komar-Samardzija, Braun, Keithley, & Quinn, 2012). Traditionally, it has been reported that women who are dissatisfied with their body size or image tend to choose diet as a weight-loss strategy (Gingras, Fitzpatrick, & McCargar, 2004), while men dissatisfied with their bodies or body image focus more on exercise and diet in order to build muscle and lose weight (Ricciardelli & McCabe, 2004). Anderson, Janes, Ziemer, and Phillips (1997) examined the relationship between satisfaction with body size and attempts to control weight in a study of African-American adults with diabetes mellitus and found that dissatisfaction with body size was significantly related to losing weight. Although many individuals begin PA for weight management reasons, nonetheless, body dissatisfaction may lead women to avoid PA. However, these findings have not been consistent in all studies, and body satisfaction was not always the specific focus of these studies (Green et al., 1997; Markey & Markey, 2005; Putterman & Linden, 2004).

The TTM of change of Prochaska and Di Clemente (1983) was often used to sustain motivations and target interventions based on PA in people with T2DM (Jackson, Asimakopoulou, & Scammell, 2007; Kim, Hwang, & Yoo, 2004; Kirk, Mutrie, MacIntyre, & Fisher, 2004). This model suggests that people progress through five stages: precontemplation (no intention to change behaviour in the next six months), contemplation (intention to change within six months), preparation (small or inconsistent changes), action (active involvement in behaviour for less than six months), and maintenance (sustained behaviour change for at least six months). Marcus et al. (2000) examined TTM-based PA interventions in people with Type 2 diabetes. Their findings suggest that people who received a TTM intervention were more likely to increase their PA levels over a six-month period. Kim et al. (2004) further illustrated that over a three-month period, participants with Type 2 diabetes who received TTM stage-based counselling showed significant improvements in stages of change for exercise behaviour and PA levels compared with a control group. Although some studies have also shown a linear relationship between PA levels and stage of change (Calfras, Long, & Sallis, 1996; Kirk, Mutrie, MacIntyre, & Fisher, 2003; Marcus et al., 2000), others have not (Naylor, Simmonds, Riddoch, Velleman, & Turton, 1999; Norris, Grothaus, & Buchner, 2000).

While the TTM has been validated in young- and middle-aged populations (Berry, Naylor, & Wharf-Higgins, 2005; Fahrenwald & Walker, 2003; Kim, 2007), limited research has been conducted on its application to older clinical populations with diabetes (Kirk et al., 2010). The study of Kirk et al. (2010), the only one to our knowledge conducted in a group of older adults with diabetes or CVD, examines the contribution of exercise self-efficacy and supports the theoretical predictions of the TTM about levels of PA. This study did not examine the contribution of body satisfaction to predict levels of PA.

Based on these premises, the aim of this study was to investigate the relationships between self-reported PA and exercise self-efficacy and body satisfaction in a sample of older adults with Type 2 diabetes classified in different stages of change. Particularly, it is hypothesized that:

(a) there are significant differences on exercise self-efficacy and body satisfaction based on stages of change behaviour and gender that approximate linear trends and (b) body satisfaction, stages of change, age, and duration of diabetes affect levels of PA, over and above exercise self-efficacy.

## Method

### Participants and procedures

Three hundred and eight (172M, 136F, mean age 65.24 ± 8.31 years) participants with Type 2 diabetes (diabetes duration 9.47 ± 8.21 years) were recruited from Centre of Diabetology, San Giovanni University Hospital, Cagliari (Italy). The diabetes mellitus was controlled by insulin (*n* = 96), diet (*n* = 31), oral hypoglycaemic agents (*n* = 164), or other combination (*n* = 17). Participants completed validated questionnaires. Data collection occurred individually in a quiet room. Before data collection, written informed consent was obtained from all participants.

### Measures

#### Participants’ characteristics

Participants' characteristics have included gender, age, treatment pattern (insulin, oral hypoglycaemic agents, diet, or other combination) and duration of diabetes.

*Exercise self-efficacy* was measured with the Exercise Self-Efficacy Scale (Marcus, Selby, Niaura, Rossi, 1992) which assesses an individual's confidence to participate in PA during five circumstances (e.g. “I am in a bad mood” and “It is raining or snowing”).

A continuum 0–100 was used, where 0 indicates being not at all confident and 100 being very confident. Cronbach's alpha reliability coefficient for the overall scale was = 0.80.

*Body satisfaction* was measured with the Silhouette-Matching Task (SMT) (Marsh & Roche, 1996). The SMT asks participants to match themselves to a series of nine body silhouette images that vary from very thin to very fat (different series are presented for males and females) in relation to actual and ideal body image rating. The discrepancies between the two ratings yield information regarding the participants' satisfaction with their body image. Cronbach's alpha reliability coefficient for the overall scale was = 0.94.

*Stage of change for* PA was assessed by asking participants to read a definition of regular PA (Marcus, Rossi, Selby, Niaura, & Abrams, 1992). Participants indicated which of five statements, each representing a stage of change, described their current PA status. The stages were defined as follows: (1) precontemplation – not regularly physically active and having no intention to become active in the next six months; (2) contemplation – not regularly physically active but thinking about starting in the next six months; (3) preparation – doing some PA but not enough to meet the description of regular PA; (4) action – regularly physically active but only began in the last six months; and (5) maintenance – regularly physically active for more than six months.

PA was assessed by asking participants to agree with the definition of regular PA of the American College of Sports Medicine/Centers for Disease Control and Prevention and indicating accordingly their weekly duration of physical activity (minutes).

### Data analysis

Data were analysed using SPSS version 19. Two cases were removed from subsequent analysis: one outlier reporting nine times the mean value of PA and another case showing a missing value in body satisfaction scale. All variables were checked about univariate and multivariate normality. Since data have shown a normal distribution, parametric analyses were used. Two separated analyses of variance were performed, using stages of change as IV and exercise self-efficacy and body satisfaction, respectively as dependent variables (DVs). One trend analysis was performed using stages of change as IV and exercise self-efficacy as DV. One *post hoc* analysis was conducted with Tukey's honestly significant difference (HSD) test using stages of change as IV and body satisfaction as DV.

Two independent *t*-tests were performed to assess gender differences in exercise self-efficacy and body satisfaction. Hierarchical regression analysis was used to predict levels of PA. The analysis considered exercise self-efficacy, body satisfaction, stage of change behaviours, age, and duration of diabetes as predictors which were entered one at time to assess their specific contribution on levels of PA. The significance level was set at *p* < .05.

## Results

Table 1 shows the distribution of participants into stages of change. The differences in scores across stages are shown in Table 2.

**Table 1.** Distribution of participants in each stage of physical behaviour change.

Stage of change	Number of participants (*n* = 306)
Precontemplation	32
Contemplation	62
Preparation	83
Action	17
Maintenance	112

**Table 2.** Mean levels of physical activity, scores of exercise self-efficacy, and body satisfaction by stage of PA behaviour change.

Measures	Precontemplation	Contemplation	Preparation	Action	Maintenance	*p*-Value	Partial eta^2^
PA (min/week)	0.00 (0.00)	38.03 (102.31)	159.76 (139.06)	285.88 (115.27)	372.59 (182.12)	.000	0.515
Exercise self-efficacy	21.63 (20.69)	43.14 (23.63)	45.36 (18.87)	65.06 (22.00)	68.78 (24.54)	.000	0.324
Fatigue	15.94 (26.23)	41.77 (32.98)	44.94 (28.82)	63.53 (29.14)	69.20 (29.97)	.000	0.245
Bad mood	24.06 (32.19)	48.39 (37.30)	54.46 (28.90)	71.76 (28.34)	76.05 (32.72)	.000	0.208
Lack of time	14.06 (23.71)	29.79 (32.27)	29.58 (30.24)	56.47 (31.21)	59.22 (38.54)	.000	0.198
Holiday	32.81 (32.72)	59.84 (36.59)	62.53 (36.18)	69.41 (35.08)	72.21 (36.44)	.000	0.093
Bad weather	21.25 (29.46)	35.89 (35.41)	35.30 (28.55)	64.12 (33.74)	65.07 (35.95)	.000	0.198
Body satisfaction	2.28 (2.70)	3.19 (2.25)	2.47 (2.59)	1.94 (2.16)	1.79 (2.34)	.008	0.045

**Figure 1.**
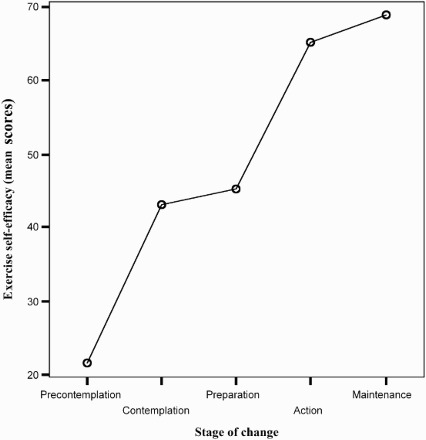
Relationship between exercise self-efficacy and stage of PA behaviour change.

The total exercise self-efficacy showed significant differences between stages, *F*(4, 301) = 36.08, *p* < .001, that approximate a linear trend *F*(1, 301) = 135.98, *p* < .001 (Figure 1).

**Figure 2.**
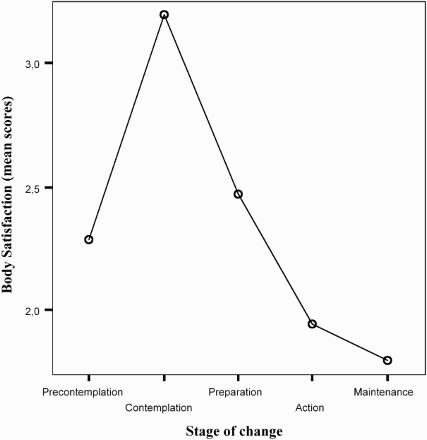
Relationship between body satisfaction and stage of PA behaviour change.

The body satisfaction presented an inverted U shape: participants in contemplation stage have shown less body satisfaction (highest scores of discrepancy) than others, *F*(4, 301) = 3.51, *p* = .008. *Post hoc* analyses conducted with Tukey's HSD have shown that the only significant difference was between preparation and maintenance (*p* < .05) (Figure 2).

Before testing gender differences in exercise self-efficacy and body satisfaction, we have collapsed patients in the action stage (*N* = 17), with the maintenance group (*N* = 112) that shows almost the same mean values in both variables.

*T*-test analyses show no gender differences in exercise self-efficacy and body satisfaction.

*Hierarchical regression analysis* was used to determine if body satisfaction, stage of change, age, and duration of diabetes improve prediction of PA levels, over and above exercise self-efficacy. Table 3 displays the correlations between the variables.

**Table 3.** The correlation matrix between variables.

Variables	PA minutes (DV)	Exercise self-efficacy	Body satisfaction	Stage of change	Age
Exercise self-efficacy	0.48***				
Body satisfaction	−0.15**	−0.10			
Stage of change	0.71***	0.56***	−0.16**		
Age	0.01	0.04	−0.07	−0.07	
Duration of diabetes	−0.05	−0.04	−0.01	−0.03	−0.05

***p < .001.

**p < .01.

A hierarchical regression analysis showed that exercise self-efficacy (*β* = 0.49, *p* < .001) accounted for 24% of the variance in PA levels, *F*(1, 303) = 95.14, *p* < .001. The addition of body satisfaction (*β* = −0.09, *p* = .06) added 1% of the variance explained in PA levels, Δ*F*(1, 302) = 3.55, *p* = .06. The addition of stage of change (*β* = 0.63, *p* < .001) was statistically significant and added 27% of the variance explained in PA levels, Δ*F*(1, 301) = 170.02, *p* < .001. Neither age nor duration of diabetes has made a significant contribution. In summary, only exercise self-efficacy and stage of change were determinants of PA levels explaining 51% of its variance.

## Discussion

To our knowledge, this is the first study examining the relationship between exercise self-efficacy, body satisfaction, and PA in different stages of PA behaviour change in older adults with T2DM.

This study reveals a positive relationship between stages of change and exercise self-efficacy, according to several authors (Marcus, Eaton, Rossi, & Harlow, 1994; Reed, 1999). The trend analysis shows that exercise self-efficacy increases linearly from precontemplation to maintenance stages. No significant gender differences in exercise self-efficacy are found.

These results support previous researches that demonstrated that personal and environmental barriers are associated with failure to participate or maintain PA (Dutton, Johnson, Whitehead, Bodenlos, & Brantley, 2005; Egan et al., 2013). These results differ from those reported by Kirk et al. (2010) that have shown a significant difference only from contemplation to maintenance stages. It should be noted that the Kirk et al. study was conducted on older adults with diabetes or CVD: the presence of these additional issues could modify the strength and the shape of the relationship between self-efficacy and stage of PA behaviour change. These finding partially support a previous statement reported by Marshall and Biddle (2001), suggesting that some transitions are more significant than others in the growth of exercise self-efficacy. More longitudinal studies are needed to verify this statement.

Body satisfaction is weakly related to the stage of PA behaviour change. In this study, participants in the maintenance stage show more body satisfaction than in other stages (Johnson, Fallon, Harris, & Burton, 2013). The participants in contemplation stage show more body dissatisfaction than others (higher discrepancy scores): this finding appears interesting to target PA interventions for older adults with T2DM. Although some studies have reported gender differences (Gingras et al., 2004; Ricciardelli & McCabe, 2004), no significant differences between males and females were found in body satisfaction in this study. Further longitudinal studies are necessary to analyse these findings.

This study takes into account the predictive value of exercise self-efficacy, body satisfaction, stage behaviour change, age, and duration of diabetes on PA levels in older adults with T2DM.

Previous research demonstrates that T2DM individuals manifest generally low levels of PA (Morrato, Hill, Wyatt, Ghushchyan, & Sullivan, 2007), and lower levels of self-efficacy for PA compared to other clinical samples and general population (Grace, Barry-Bianchi, Stewart, Rukholm, & Nolan, 2006; Plotnikoff, Brez, & Brunet, 2003). The results confirm that exercise self-efficacy plays an important role as predictor of PA levels. Self-efficacy is often reported as one of the most consistent predictors of PA levels (Aljasem et al., 2001; Delahanty, Conroy, & Nathan, 2006; Dutton et al., 2009), also in patients with Type 2 diabetes (Delahanty et al., 2006; Grace et al., 2006; Kim, McEwen, Kieffer, Herman, & Piette, 2008; Nelson, McFarland, & Reiber, 2007; Plotnikoff, Brez, & Hotz, 2000).

Some empirical researches have demonstrated that body satisfaction facilitates PA (Campbell & Hausenblas, 2009; Hausenblas & Fallon, 2006; Huberty, Ransdell, & Sidman, 2008; Kruger, Lee, Ainsworth, & Macera, 2008; Millstein, Carlson, & Fulton, 2008; Trost, Owen, & Bauman, 2002; Wilcox, Richter, & Henderson, 2002), while others have shown that body satisfaction represents a barrier to exercise (Leary, 1992; Martin, Leary, & O'Brien, 2001). Some of these research studied only females or did not analyse separately for males and females. Some did not control body mass index (BMI). Our results indicate that body satisfaction is a weak predictor of PA levels, compared to self-efficacy and stages of change. This study provides an initial contribution on the role of body satisfaction to promote PA levels in older adults with T2DM, but suffers from the limitations cited above. Further studies should control gender differences and BMI to improve the predictability of body satisfaction. This study gives further support to the hypothesis of a linear relationship between stages of change and PA levels. These stages of change show a relevant and significant contribution to the prediction of PA, and appear to be a crucial component in targeting PA interventions. However, this study does not include analysis of the processes of PA behaviour change. As Marshall and Biddle (2001) demonstrated, the frequency of use of the processes of self-liberation, counter-conditioning, and reinforcement management is particularly important for encouraging T2DM adults to adopt active lifestyles, namely facilitating the transition from preparation to action stages. Further studies must evaluate the relationship between these processes and the stages of PA behaviour change in adults with T2DM.

We acknowledge that the study has some limitations. First, participants in this study consist in a convenience sample recruited from one diabetes centre, which limits the generalizability of the study findings. Another limitation includes reliance on self-report rather than objective measures of PA (e.g. pedometers and accelerometers). Although self-reported measures have generally shown a good proxy of PA, more objective measures of energy expenditure should be included in the continuation of the studies (e.g. armband).

Considering a clinical sample of older adults short measures of psychological variables were used, which are subject to potential response bias. Extraneous variables such as the participants' discomfort with some questions, fatigue, decreased cognition, a lack of understanding of the questions, and response bias may have influenced the self-report results and limited the validity of our study. Lastly, the present study does not examine clinical variables (i.e. Glycosylated hemoglobin [HbA1c] BMI) and their role for adherence to PA.

Further studies are necessary to maintain potentially confounding variables under control (i.e. gender) and to relate psychological variables with clinical indicators. These researches should include longitudinal intervention studies to establish the direction of founded relationships. However, our study gives a first contribution on the relevance that exercise self-efficacy and body satisfaction assume to sustain PA in older adults with T2DM, targeted by means of the stage of change for PA behaviour.
